# Individualised sensory intervention to improve quality of life in people with dementia and their companions (SENSE-Cog trial): study protocol for a randomised controlled trial

**DOI:** 10.1186/s13063-018-2973-0

**Published:** 2019-01-25

**Authors:** Jemma Regan, Eric Frison, Fidéline Collin, Piers Dawes, Mark Hann, Ines Himmelsbach, Emma Hooper, David Reeves, Zoe Simkin, Chryssoula Thodi, Fan Yang, Iracema Leroi, for the SENSE-Cog Trial Development Team, Harvey Abrams, Nathalie Chaghil-Boissière, Pavlina Charalambous, Fidéline Collin, Fofi Constantinidou, Eric Frison, Camille Gilbert, Mark Hann, Catherine Helmer, Emma Hooper, Francine Jury, Evangelia Kontogianni, Brian Lawlor, Iracema Leroi, Charly Matard, Susana Montecelo, Sarah Marie, Antonios Politis, Otilia Postea, Jemma Regan, David Reeves, David Renaud, Zoe Simkin, Monique Termote, Chryssoula Thodi, Lucas Wolski

**Affiliations:** 1 Division of Neuroscience and Experimental Psychology, University of Manchester and the Manchester Academic Health Sciences Centre, Oxford Road, Manchester, M13 9PL UK; 2 University of Bordeaux, INSERM, EUCLID/F-CRIN Clinical Trials Platform, Bordeaux Population Health Center, CHU Bordeaux, F-33000 Bordeaux, France; 3 Manchester Centre for Audiology and Deafness (ManCAD) University of Manchester and the Manchester Academic Health Sciences Centre, Oxford Road, Manchester, M13 9PL UK; 4 Division of Population Health, Health Services Research & Primary Care, University of Manchester, Grafton Street, Manchester, M12 9NT UK; 5 Institute of Applied Research, Development and Continuing Education, Catholic University of Applied Sciences, Karlstraße 63, Freiburg, 79104 Germany; 6 School of Sciences, Department of Health Sciences, European University Cyprus, Diogenis Street, Nicosia, 2404 Cyprus

**Keywords:** Dementia, Hearing, Vision, Sensory intervention, Quality of life, Europe, Psychosocial intervention

## Abstract

**Background:**

Hearing and vision impairments are highly prevalent in people with dementia and may have a negative impact on quality of life and other dementia-related outcomes. Intervening to optimise sensory impairment and support sensory function may be a means of improving dementia-related outcomes. The SENSE-Cog trial will test whether a home-based multi-part sensory intervention is effective in improving quality of life and other key outcomes in people with dementia and hearing or vision problems (or both) and their companions.

**Methods:**

This is an European, multi-centre, observer-blind, pragmatic, randomised controlled trial. Three hundred fifty four people with dementia and hearing or vision impairment (or both) and their companions will be randomly assigned to receive either “care as usual” or a multi-component sensory intervention including assessment and correction of hearing or vision impairments (or both), home-based (maximum 10 visits over 18 weeks), therapist-delivered sensory support (that is, adherence to devices; improving the sensory environment (that is, lighting), communication training, and sign-posting to other support agencies). Change from baseline to intervention end (18 weeks) and post-intervention (36 weeks) will be compared between the two arms in the following outcomes: quality of life (primary endpoint), sensory and cognitive functional ability, relationships, mental well-being, health resource utilisation and cost-effectiveness.

**Discussion:**

This is one of two articles outlining the SENSE-Cog trial. Here, we describe the protocol for the effectiveness of the SENSE-Cog intervention. A parallel and complementary process evaluation will be described elsewhere. If the SENSE-Cog trial demonstrates that the sensory intervention improves outcomes in dementia, we will make a toolkit of training materials, resources and information available to health and social care providers to implement the intervention in routine practice. This will be a significant contribution to the therapeutic management of people with dementia and sensory impairment.

**Trial registration:**

ISRCTN (Trial ID: ISRCTN17056211) on 19 February 2018.

**Electronic supplementary material:**

The online version of this article (10.1186/s13063-018-2973-0) contains supplementary material, which is available to authorized users.

## Background

The prevalence of dementia in Europe is high and rising; nearly 10.5 million Europeans are currently diagnosed with dementia [1]. Age-acquired hearing impairment or age-acquired vision impairment or both affect one in three Europeans [2]. People with dementia (PwD) are more likely to experience sight loss [3] and are more likely to self-report hearing difficulties [4] than their cognitively healthy, senior counterparts. Thus, the likelihood of co-morbid vision or hearing impairment (or both) is a very real possibility for PwD in Europe [5].

Later-life peripheral hearing loss has been newly identified as a potential risk factor for dementia [6] and may be modifiable through the use of hearing aids [7, 8], although the evidence for this is still accruing [9]. Furthermore, improving or reversing sensory impairment in PwD is challenging. Specifically, whereas the rate of self-reported impairment in PwD is high, the diagnostic rate of hearing and vision impairments is low [10]. Corrective equipment for vision [3] and hearing [11] is not always prescribed when required, and if it is prescribed, adherence is often inconsistent [12]. Thus, in PwD with concurrent sensory problems, simply correcting the impairment may be insufficient to improve outcomes.

In cognitively healthy older people, training and support interventions to improve hearing aid adherence [13] and home-based assessments to enhance the uptake of glasses have been successfully implemented [14]. Moreover, it has been demonstrated that optimising hearing can positively affect mental status [15] and cognitive function [16]. Unfortunately, these studies have not addressed similar questions in people who have been diagnosed with dementia [11]. Despite this, there is preliminary evidence that sensory remediation in dementia is effective in reducing personal and social difficulties when vision is improved [17], decreasing behavioural and psychological symptoms of dementia with improved hearing [18], reducing depression [19], and improving cognition and mood [20]. Importantly, to be effective, treatment should be introduced at an early stage in dementia [1] and should be tailored to the specific care needs of each individual [10]. For example, when clinical sensory assessments with PwD are conducted, existing vision assessments should be adapted to account for fluctuating mental capacity, decreased executive functioning, and reduced decision-making ability [3].

Optimising hearing and vision per se may not be sufficient to improve outcomes for PwD. To extend a hearing and vision intervention in PwD beyond just a sensory assessment and fitting of corrective devices, further components need to be introduced. These could entail support from a trained therapist (that is, a “sensory support therapist”, or SST), aspects of behavioural change, and greater access to support services. Implementing behavioural change can be difficult and evidence demonstrates that behavioural changes, when attempted, may not be sustained unless key underlying elements are addressed [21]. There is evidence that psychosocial interventions, introduced at an early stage of dementia, may benefit quality of life (QoL) and other key dementia-related outcomes [22–25]. Over the course of 18 months, guided by the UK Medical Research Council’s framework for developing complex interventions, we used the process of “intervention mapping” [26] to develop the sensory intervention (SI) [26]. The SI was initially field-trialled in the UK, France and Cyprus [27] and was subsequently refined for full-scale trialling across five European sites described in this article. The SI includes the following: (1) assessment of hearing and vision function, (2) correction of hearing and vision impairments, and (3) a home-based psycho-social intervention, encompassing communication training, environmental modification, and sign-posting to further support services, delivered by a trained therapist. To test the effectiveness of the intervention to improve QoL in PwD with hearing or vision impairment (or both) and their companions, we designed the SENSE-Cog trial, which is a multi-centre, observer-blind, pragmatic, randomised controlled study comparing the SI with care as usual (CAU). Secondary objectives will investigate the impact of the intervention on sensory and cognitive functional ability, the relationship of the PwD and their companion, mental well-being, and companion outcomes. We will also investigate health resource utilisation following the intervention and estimate cost-effectiveness of the intervention.

The SENSE-Cog randomised controlled trial (RCT) is outlined in two parts. The present article introduces the protocol for evaluating the effectiveness of SI compared with CAU. A separate article will outline the protocol for the process evaluation, assessing delivery, contextual issues and causal mechanisms of the SI.

### Research question

The SENSE-Cog trial aims to address the following research question: Does SI (correction of sensory impairment combined with sensory support) improve the QoL of PwD and their companions, across Europe?

The SENSE-Cog trial aims to test the following hypotheses:

- the application of SI will enhance QoL for PwD and sensory impairment;
- the SI will improve functional ability for the PwD (defined by cognition-, hearing- and vision-related activities of daily living - ADLs) and mental well-being (defined by improved global cognitive ability, self-efficacy, relationship with the companion, and reduced behavioural disturbances);
- the SI will improve mental well-being, improve the relationship with the PwD, and reduce burden and stress (as defined by improved companion experience, well-being and anxiety and depression) for companions of PwD.

## Methods/design

This is a 36-week, multi-centre, randomised, controlled, pragmatic, parallel-group, observer-blind, superiority trial comparing the effectiveness of individualised SI with CAU on QoL and other dementia-related outcomes in PwD with hearing or vision impairment (or both) and their companions in five European sites. Participants will be randomly assigned after baseline to either the SI group or CAU group in a 1:1 ratio. The SI is composed of three parts delivered over the course of 18 weeks: (1) assessment of sensory impairment, (2) correction of sensory impairment, and (3) SST weekly home-based visits (maximum of 10). A subsample of 60 dyads (PwD and their companion) in the SI group will also complete a qualitative interview within 2 weeks of the end of the SI.

### Participant selection

Participants will be recruited in “dyads” (that is, a PwD and a companion: relative or friend) in accordance with the criteria outlined below. Of note, a PwD with hearing or vision impairment (or both) cannot participate if the companion is ineligible or unwilling to participate.

#### Person with dementia inclusion criteria

- Is at least 60 years old;
- is diagnosed with dementia in accordance with ICD-10 (10th revision of the International Statistical Classification of Diseases and Related Health Problems) criteria because of the following conditions: Alzheimer’s disease (AD) (in accordance with NINCDS-ADRDA [28] criteria) or vascular dementia (VAD) (in accordance with NINDS-AIREN [29] criteria) or “mixed” dementia (AD + VAD);
- has dementia in the mild to moderate stage, as indicated by a Montreal Cognitive Assessment (MoCA) [30] score of at least 10;
- if taking cognitive enhancing medication (that is, cholinesterase inhibitors or memantine), this must be on a stable, unchanged dose for at least 4 weeks prior to screening;
- has adult-acquired hearing or vision impairment (or both) defined by
  - *vision impairment*: defined by the presence of
    - presenting binocular visual acuity of not more than 6/9.5 and greater than 6/60 in Snellen metric (or at least + 0.2 logMAR [75 EDTRS Score] and less than + 1.0 logMAR [35 EDTRS Score]) using the Portable Eye Examination Kit (PEEK) vision tool [31] and
  - visual field greater than 10° using confrontation visual field test [32]

and/or

- *Hearing impairment*: defined by a bilateral hearing difficulty, indicated by failure of a pure tone hearing screening test in both ears, defined by hearing worse than 35 dBHL at 1000 Hz and above in the better ear, using the HearCheck device [33];

- lives in an ordinary community dwelling (including sheltered and very sheltered accommodation);
- is willing to accept SI;
- has a companion who fulfils the criteria below and is willing to participate in the study;
- has the capacity to provide informed consent to participate in the study or, if lacking that capacity, has a nominated consultee to provide consent on their behalf;
- speaks and understands the language of the intervention delivery, as determined by the investigator;
- is affiliated with a social security system (for France).

#### Person with dementia exclusion criteria

- Has an unstable, acute or current psychiatric or physical condition severe enough to prevent them from participating in the study, as determined by the investigator;
- has complete blindness or severe visual impairment (category 2 and more on ICD-10) [34] or deafness (profound hearing loss) that will prevent them from following study procedures;
- is currently participating in any other trial of a potentially cognition-enhancing intervention, excluding marketed cognition-enhancing medication;
- has scheduled or urgent treatment or intervention for hearing or vision impairment (that is, cataract operation already scheduled or treatment for macular degeneration is needed);
- is unable to read and write.

#### Companion inclusion criteria

- Is at least 18 years old;
- is an informal caregiver (where providing care is not the person’s primary paid role), such as a significant other of the PwD (for example, a family member or close friend), who is either co-resident or in regular contact (on at least a weekly basis);
- is willing to participate in the study;
- speaks and understands the language of intervention delivery, as determined by the investigator;
- is affiliated with a social security system (for France).

#### Companion exclusion criteria

- Has an unstable, acute or current psychiatric or physical condition severe enough to prevent them from participating in the study, as determined by the investigator;
- is unable to read and write.

### Sensory intervention

The three parts of the SI are described as follows:

- Stage 1: Assessment of sensory impairment

A full vision or hearing assessment (or both) will be undertaken by an audiologist, optometrist or ophthalmologist, in accordance with clinically regulated, standardised procedures (Table 1), in the participant’s home or in the clinic within 8 weeks after randomisation. Should medical management of cataracts or macular degeneration be identified, participants will remain in the study and the SI will be offered within an 18-week period, which does not interfere with scheduled surgery.

**Table 1:** Clinical audiology and ophthalmology examination procedures

Audiology examination	Ophthalmological examination
Otoscopy: examination of the pinna (outer ear) and external auditory meatus (ear canal) using British Society of Audiology (BSA)-recommended procedure for otoscopy (British Society of Audiology, 2010)	Observation of eyes and adnexae for any pathology, visual field testing (using confrontation test and amsler grid for screening major visual field deficits), and intraocular pressure measures to detect any ocular pathology
Ambient noise: background noise checks should be made prior to and during audiometric testing to ensure that noise levels do not go over the recommended level of 35 dBA as stated in the BSA-recommended procedure (40 dBA maximum) for pure tone air conduction and bone conduction threshold audiometry with and without masking.	Current optical correction: determination of lens type and power with associated distance and near visual acuity, used as baseline visual performance
Pure tone audiometry: air conduction and bone conduction according to BSA-recommended procedures for pure tone air conduction and bone conduction threshold audiometry	Visual needs: Identification of main activities with associated distance and global light sensitivity to make refraction at appropriate distance; recommendation of any adaptive equipment to cover unmet visual needs
Glasgow Hearing Aid Benefit Profile (GHABP) [41]	Visual function evaluation: ascertainment of subjective refraction (or objective when, owing to factors such as poor cooperation, subjective is not possible) with associated visual acuity, contrast sensitivity, and binocular vision

- Stage 2: Correction of sensory impairment

Glasses or hearing aids (or both) will be prescribed, administered and fitted to participants, according to their needs, by vision specialists (optometrist, ophthalmologist or optician) and audiologists, respectively, in the participant’s home or in the clinic within 6 weeks after full assessment (stage 1). Essilor International [35] will provide the glasses lenses and yellow filters for the study. Starkey Hearing Technologies [36] will provide the hearing aids and two pocket talkers (http://www.starkey.co.uk/hearing-aids/hearing-amplifiers) per site. The hearing devices used for this trial will be behind-the-ear (BTE)-style hearing instruments (specifically, Starkey Muse i2400 Mini BTEs in silver). Supplementary sensory devices (lamps and glasses straps) may be supplied according to participant needs by the SST throughout the SI (Table 2).

**Table 2:** SENSE-Cog Randomised Controlled Trial Sensory Intervention sensory devices to be supplied, who pays costs and duration

Device	Supplier	Cost	Duration
Hearing aid: Muse i2400 Mini Behind the ear	Starkey Hearing Technologies	Free to participant	Participant keeps during and after study
Personal listening amplifier	Accredited supplier such as Mini Tech, Pocket Talker, or equivalent	Free to participant	Return after study and option to purchase at participant’s own expense
Additional auditory or visual equipment as advised by the sensory support therapist	Starkey Hearing Technologies or other specialist suppliers	Participant’s own expense	Participant keeps during and after study
Glasses lenses (including yellow filters if needed)	Essillor International	Free to participant	Participant keeps during and after study
Glasses frames (participant choice)	Local optician	Free basic frames or other frames at participant’s own expense	Participant keeps during and after study
Lamp	Any supplier provided that the required criteria are met	Free to participant	Return after study and option to purchase at participant’s own expense
Glasses straps	Croakies or equivalent supplier	Free to participant	Participant keeps during and after study

- Stage 3: Sensory support

**Fig. 1:**
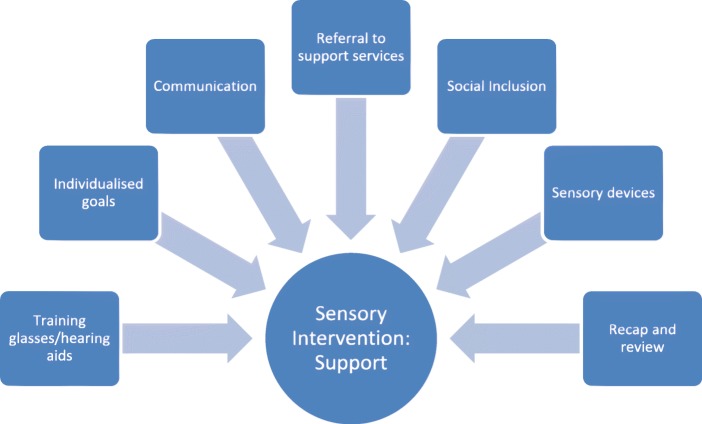
The SENSE-Cog Randomised Controlled Trial Sensory Intervention: Support components delivered by the sensory support therapist

The SST will support participants with the following '*primary*' (all participants receive) and '*secondary*' (received if needed by individual) components across a maximum of 10 visits over 18 weeks after randomisation (see Fig. 1):

#### Continuous training in correct use of sensory devices (primary component)

The SST will support correct wear and care of participant’s glasses and hearing aids (for example, cleaning and storage, battery changing, and frequency of use). The Hearing Aid Skills and Knowledge (HASK) test [37] and SENSE-Cog Glasses Skills and knowledge test for vision alongside a SENSE-Cog functional assessment (a non-standardised assessment developed by the research team) [27] will be completed to monitor participant abilities to manage their sensory equipment.

#### Supporting progress towards individualised goals (primary component)

The SST will incorporate the results of the hearing, vision and daily living functional assessments to set individual goals by using the Bangor Goal Setting Inventory [38]. A maximum of three goals will be set with participants and at least one of these goals will explicitly include the use of their corrective sensory device(s). Goals will typically be revisited on a weekly basis, and the SST will explore facilitators, barriers and resources to the goals and introduce skills and strategies to support progress. Goal attainment and performance will be re-rated by the participant and their companion on completion of the SI.

#### Enhancing communication between the PwD and their companion (primary component)

The SST will work with the participant dyads on improving communication by using the SENSE-Cog Communications Manual. This information has been adapted from existing, evidence-based resources relating to sight/hearing loss and dementia to provide guidance and strategies to enhance communication in different settings. Copies of pre-existing materials such as leaflets will be provided to the participants.

#### Accessing relevant support services (such as psychological services) by referral (secondary component)

This may include psychological services, geriatric psychiatry services, falls clinics, or other health or social care services beyond the remit of the SENSE-Cog study. The SST will identify the participant’s requirements through the functional assessments and goal-setting exercise.

#### Fostering social inclusion through hobbies, interests and groups (secondary component)

The SST will provide information and guidance to participants about opportunities to develop their own hobbies and interests or attend local groups in line with participant goals.

#### Guidance about supplementary sensory devices (secondary component)

Participants with vision loss will be offered the opportunity to trial a lamp to assist with low-vision for the duration of the intervention. The lamp spec must provide an illuminance on a work surface at 30 cm of at least 500 lx and ideally 1000 lx. Participants will be provided with glasses straps if required. The SST will explore whether additional sensory devices such as a media streaming device [39] or Hearing Amplifier (http://www.starkey.co.uk/hearing-aids/hearing-amplifiers) would promote a sensory-conducive home environment. Some of these devices will be loaned to participants for the duration of the intervention and information provided about where they may purchase them post-SI at their discretion (Table 2).

#### Re-cap and review visits

If a dyad addresses all SI components prior to the end of 18 weeks, any remaining weeks will involve further recap and review of progress, up to a maximum of 10 SST visits.

### Care as usual

The CAU group provides a comparison with the SI group. CAU participants will receive no additional intervention other than hearing and vision screening assessment. CAU participants will be informed of any suspected hearing or visual impairment (or both) identified on screening and information sheets provided about where they may access further support through their general practitioner or standard routes of referral. Thus, we except a small increase in subsequent diagnosis of sensory impairment compared with usual care but no effect on prescription of appropriate correction and adherence. Thus, effect estimate of the SI compared with this CAU group should be slightly decreased and conservative regarding type I error rate. Differences in access of health services between the intervention group and CAU group will be captured by the health economic evaluation measure (Resource Utilization in Dementia-Lite, or RUD-Lite) [40].

### Recruitment

There will be several routes for participant recruitment, depending on the specific study site. In the UK (Manchester), the National Health Service (mental health or memory assessment services) will be the first-line sources for recruitment. Other routes to participation will include the on-line national dementia clinical research portal, ‘Join Dementia Research’ (www.joindementiaresearch.nihr.ac.uk), alongside referral from local primary care clinics, dementia support groups and the Alzheimer Society. In France (Nice), recruitment will be from the *Centre Mémoire de Ressource et de Recherche*’s clinical database and local primary care, neurology and geriatric medicine clinicians. In Greece (Athens), participants will be recruited from organised dementia care centres, the Geriatric Psychiatry Outpatient Memory Clinic, the Nestor Psychogeriatric Association and the Athens Alzheimer Association. In Dublin, recruitment will be from the memory clinic at the Mercer’s Institute for Successful Ageing, St. James’s Hospital. Finally, in Cyprus (Nicosia), participants will be identified from dementia care centres, mental health services, the Ministry of Health and private practice.

### Informed consent

This procedure will be in accordance with the national guidance regarding informed consent and clinical research with individuals who lack capacity in each of the participating countries. Prior to obtaining written consent, the researcher will ensure that the person is fully informed about the research and take time to answer any questions. Informed written consent will be obtained by the researcher at the participant’s home or in clinic before any study-specific procedure for screening. All researchers will be fully trained in Good Clinical Practice (GCP) and mental capacity assessment skills and follow national guidance in their respective countries, such as the Mental Capacity Act (2005) [41] in the UK. If a person lacks capacity, a consultee—either a personal (family/friend) or nominated (professional)—will be asked to deem whether it is in the PwD’s best interests to participate.

### Sample size

The trial is powered to detect a standardised effect size of 0.267 (equivalent to a 4-point change) on the Dementia Quality of Life (DEMQOL) [42] and assuming a standard deviation of 15 points in DEMQOL [42] scores. In this population, this effect size is equivalent to the smallest change that could be considered clinically meaningful. Assuming a correlation of 0.6 between baseline and 36-week follow-up DEMQOL scores [42] and an attrition rate of 20% at follow-up (a conservative estimate based on the 12%–15% rates observed by Wenborn *et al*., 2008 [43]), the trial will need to recruit 354 participant dyads at baseline (177 per arm) in order to achieve 80% power to detect the aforementioned effect size at the two-sided 5% level of significance.

### Outcome measures

#### Primary outcome

The primary outcome will be QoL of the PwD, as rated by the PwD directly, using DEMQOL [42], at week 36 (W36). DEMQOL is a 29-item interviewer-administered, self-report questionnaire with good psychometric properties in persons with mild to moderate dementia (good internal consistency and test–retest reliability and moderate validity) (see Table 3) (Additional file 1).

**Table 3:**
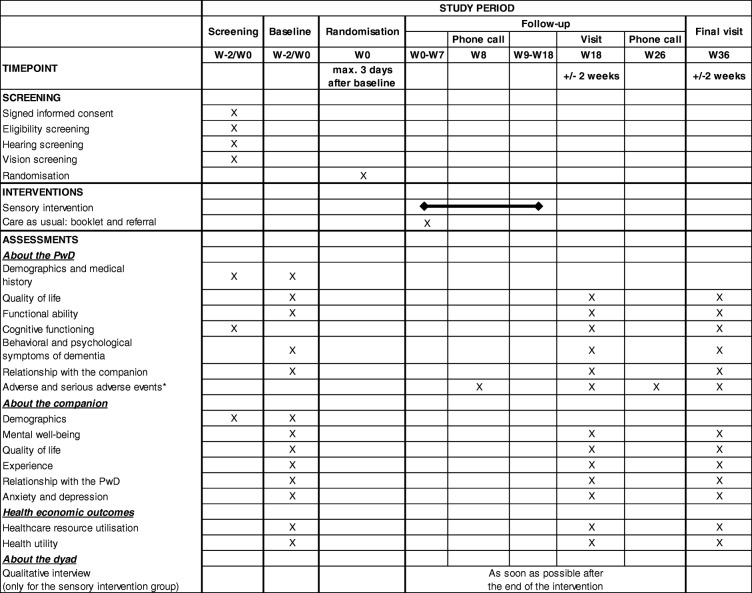
Schedule of the SENSE-Cog randomised controlled trial, according to SPIRIT checklist. * Also during sensory support therapist visits for those allocated to the sensory intervention group. Abbreviations: PwD participant with dementia, W week

#### Secondary outcomes

For the person with dementia:

- QoL assessed by DEMQOL at W18
- QoL assessed by DEMQOL Proxy, at W18 and W36, rated by the companion
- scores of the following measures at W18 and W36:
  - ○ functional ability, assessed by the following scales:
    - ▪ Bristol Activities of Daily Living scale [44];
    - ▪ Veterans Affairs Low Vision-Visual Functioning Questionnaire [45];
    - ▪ Veterans Affairs Low Vision-Visual Functioning Questionnaire Spousal rating [45];
    - ▪ Hearing Handicap Inventory for the Elderly [46];
    - ▪ Hearing Handicap Inventory for the Elderly Spousal rating [47].
  - ○ global cognitive functioning, using the MoCA scale [30].
  - ○ behavioural and psychological symptoms of dementia, assessed by the 12-item Neuropsychiatric Inventory [48].
  - ○ relationship with companion, assessed by the Relationship Satisfaction Scale [49].

For the companion:

- mental well-being and QoL using the 12-item General Health Questionnaire [50] and health utility using the Short Form-12 Health Survey [51] (Brazier and Roberts, 2004 [51]), at W36;
- scores of the following measures at W18 and W36
  - ○ companion experience, assessed by the Family Caregiving Role scale [52];
  - ○ relationship with PwD, assessed by the Relationship Satisfaction Scale [49];
  - ○ companion anxiety and depression, assessed by the Hospital Anxiety and Depression scale [53].

Health economic outcomes:

- health-care resource utilisation from baseline to W18 and W36 collected using the RUD-Lite instrument [40] with the companion;
- health utility, ascertained at baseline, W18 and W36 from:
  - ○ 5-level EuroQol 5-dimension rated by both the PwD and the companion [54];
  - ○ Short Form-12 Health Survey [51], rated by both the PwD and the companion.

### Demographics

Demographic information about the PwD and companion will be captured at screening and baseline relating to age, gender, maritial status, living status, current or former occupation, duration and type of memory problem, diagnosis date, years in formal education, date of most recent hearing and vision screen, current medication for dementia and current psycho-social interventions. This will allow screening of eligible participants and will allow analysis of the potential influence of demographic differences on outcome variables within and between sites.

### Study procedures

#### Timeline

- Start of inclusion period: first quarter of 2018;
- duration of the inclusion period: 21.5 months;
- duration of participation of each participant: 36 to 40 weeks (that is, around 9 to 10 months);
- total duration of the study: 33–34 months.

#### Initial screening visit

Cognition will be assessed by MoCA [30]. Hearing will be screened by using “HearCheck” [33] (a simple hand-held screening device). Vision will be screened by using the “PEEK Acuity App” [31] alongside the confrontation visual field test: “Can you see my hands?” [32].

#### Baseline visit

The baseline visit is conducted at the PwD’s home or clinic by a researcher. PwD and companion complete a battery of scales (around 2 h) (Table 4). The baseline visit may be conducted the same day as the screening visit (but after the screening procedures) if the PwD and the companion meet the eligibility criteria. This baseline visit may also be split into two visits (depending on the PwD or the companion’s preference). The second baseline visit must be conducted within 2 weeks of the first baseline visit.

**Table 4:** Battery of scales administered during baseline, week 18 and week 36 visits

Outcome	Administered by	Information about	Scale
QoL of PwD	Researcher to PwD	PwD	DEMQOL [42]
	Researcher to companion	PwD	DEMQOL proxy [42]
Dementia-related functional ability	Companion self-completes	PwD	BADLs [44]
Vision-related functional ability	Researcher to PwD	PwD	LV-VFQ – 20 [45]
	Researcher to companion	PwD	LV-VFQ – SP
Hearing-related functional ability	Researcher to PwD	PwD	HHIE-25 [46]
	Researcher to companion	PwD	HHIE-SP [47]
Global cognitive functioning	Researcher to PwD	PwD	MoCA (at screening) [30]
Behavioural and psychological symptoms	Researcher to companion	PwD	NPI-12 [48]
Relationship with companion	Researcher to PwD (not in presence of companion)	PwD	RSS [49]
Mental well-being of companion	Companion self-completes	Companion	GHQ-12 [50]
QoL of companion	Researcher to companion	Companion	SF-12 [51]
Companion experience	Companion self-completes	Companion	FCS [52]
Relationship with PwD	Companion self-completes	Companion	RSS [49]
Companion depression	Companion self-completes	Companion	HADS [53]
Health resource utilisation	Researcher to companion	PwD	RUD-Lite [40]
	Researcher to companion	PwD	EQ-5D-5 L Proxy [54], SF-12 Proxy [51]
	Researcher to PwD	PwD	EQ-5D-5 L [54], SF-12 [51]

*Abbreviations*: *BADLs* Bristol Activities of Daily Living, *DEMQOL* Dementia Quality of Life, *EQ-5D-5 L* 5-level EuroQol 5-dimension, *EQ-5D-5 L-P* 5-level EuroQol 5-dimension Proxy, *FCS* fluorescence correlation spectroscopy, *GHQ-12* General Health Questionnaire – 12 items, *HADS* Hospital Anxiety and Depression Scale, *HHIE-25* Hearing Handicap Inventory for the Elderly - 25 items, *HHIE-SP* Hearing Handicap Inventory for the Elderly Spousal rating – 25 items, *LV-VFQ – 20* Veterans Affairs Low*-*Vision Visual Functioning Questionnaire – 25 items, *LV-VFQ – SP* Veterans Affairs Low*-*Vision Visual Functioning Questionnaire Spousal rating – 25 items, *MoCA* Montreal Cognitive Assessment, *NPI-12* Neuropsychiatric Inventory – 12 items, *PwD* people with dementia, *QoL* quality of life, *RSS* Relationship Satisfaction Scale, *RUD-Lite* Resource Utilization in Dementia-Lite, *SF-12* Short Form-12 Health Survey

### Data protection and sharing

We will follow best practice in accordance with current UK General Data Protection Regulation (GDPR) guidance and adhere to the Guidelines for Data Management in Horizon 2020 and local guidelines for each site; no patient identifiable data (PID) will be transferred across sites. All PID will be kept separately from the anonymized data to be entered in the case report form (CRF). The University of Manchester institutional repository (Research Data Management Service) will cater for the publishing and sharing of research data. Data will be made available through the University of Manchester institutional repository (Research Data Management Service). Research data will be made available together with appropriate metadata in line with Horizon 2020 policy to enable other researchers to identify whether the data could be suitable for re-use. Published outputs will be assigned a Digital Object Identifier to reference the data in publications. Anonymised data will eventually be made open access and this will also be made clear to participants before they consent.

### Randomisation and allocation concealment

Randomisation will be performed no later than 3 days following baseline. Randomisation will be conducted through the trial electronic case report form (eCRF) [55]. One person in each site will perform randomisation. One person from the research team will be identified in each site to perform randomisation, excluding the blinded researcher who will undertake the outcome visits. Randomisation will be stratified by centre. Once participants are allocated to the eCRF, eligibility criteria (including signed informed consent) must be met to allow for randomisation online. The SST will then trigger the local procedure for sensory assessment and provision of hearing aids/glasses for those participants allocated to the SI group.

The randomisation code will be maintained by the European Clinical Trials Platform & Development (EUCLID) Coordinating Trial Unit (CTU). The blinded researcher and the statistician team will not have access to the randomisation code. To ensure allocation concealment, full details of the randomisation scheme will not be disclosed.

### Blinding

It will not be possible for participant dyads to be blinded to the allocation they receive on randomisation. Thus, each centre will have both blinded and un-blinded researchers involved with different aspects of the study. The SST will not be blinded. The outcome rater (blinded researcher) will be blinded and every attempt to maintain this blind will be made. To achieve this, when the follow-up visits are booked by the SST, the participant dyad will be reminded to make every effort not to reveal whether they had received the intervention or not. This will include keeping study-related materials (including new devices such as hearing aids, special lamps or other sensory support devices or materials) out of sight during the outcome rating visit.

During follow-up visits, an un-blinded researcher will administer MoCA [30] and HHIE (Hearing Handicap Inventory for the Elderly - 25 items) [46] with the PwD still wearing hearing aids and glasses if required. The blinded researcher will conduct the remainder of the measurement scales while the participant is wearing glasses but not hearing aids. Since most of the outcome measures will be undertaken with the companion as informant for either themselves or the PwD, this should not interfere with the integrity of the outcome measures.

For the blinded researcher, ratings will be ascertained of their perception of allocation of participant dyads to determine the strength of the allocation concealment. At W18 and W36, blinded researchers will rate a Likert-style scale [56] (completely certain, somewhat certain, some doubt, not at all certain, or complete guess) of their perception of which group the participant dyad has been assigned to. This will be analysed to ascertain the proportion of blinded researchers who are correct, incorrect or neutral for treatment allocation at W18 and W36.

### Follow-up visits

At 18 weeks (± 2 weeks) and 36 weeks (± 2 weeks), researchers will visit the PwD and their companion to complete the same battery of scales as at the baseline visit (Table 4). If a second visit is required because of fatigue, this will take place within 2 weeks following the first.

### Therapist compliance with protocol

The SST will update a logbook after each SI session to record the following: participant progress, motivation, adherence to equipment, and emotional enagement. The SST will have monthly individual and 3-monthly group supervision sessions with the lead SST, who will oversee the delivery of the intervention across the five sites. The lead SST will review the SST logbooks to discuss at each supervision, and particpant details will be anonymised.

### Participant adherence to the intervention

Adherence to the use of sensory equipment and other intervention procedures will be documented by the PwD and companion in pre-printed diaries and in the SST logbook and willl be described during the post-intervention semi-structured interview. Details of how these data will inform analysis of process measures will be detailed in the process evaluation protocol article.

### Analysis of Outcomes: Descriptive analyses

Continuous and ordinal variables will be described in terms of absolute frequency, mean, standard deviation, 95% confidence interval of the mean, median, interquartile range, and minimum and maximum. Categorical variables will be described in terms of number, proportion and 95% exact binomial confidence interval of proportion.

### Quantitative analyses: Test of the intervention effect

For estimating and testing the effect of the intervention on the primary (DEMQOL score at 36 weeks) and secondary outcomes, an *a priori* Statistical Analysis Plan (SAP) will be devised, detailing the analysis methods, outcomes, covariates, handling of missing data, standard error estimation methods and any sensitivity analyses. The SAP will be submitted to the Trial Steering Committee (TSC) for review and approval prior to the start of statistical analyses. Intention-to-treat principles will be followed, and all randomly assigned participants will be analysed according to trial arm allocation, including as far as possible those who discontinue the study, for whom follow-up data will continue to be collected wherever possible.

A separate multi-level (participants within sites) mixed-effects regression analysis will be conducted for each outcome to estimate and test the mean effect of the intervention at 18 and 36 weeks. In each case, the dependent variable will be the outcome scores at baseline, 18 and 36 weeks, and covariates will be trial arm, time point and pre-specified participant- and country-level covariates. The tests for treatment effect at 18 and 36 weeks will be based on the relevant component of the trial arm by time-point interaction. Study site will be treated as a fixed effect. The primary analysis will use complete cases only; sensitivity analyses will assess robustness of results to concerns, including non-normality (using the non-parametric bootstrap method of standard error estimation), missing values (using single or multiple imputation as appropriate) and baseline imbalance (by inclusion of unbalanced covariates). Between-site heterogeneity in treatment effects will be explored by using moderator analysis. The statistics team at Manchester University will conduct all analyses using Stata statistical software [57]. All statistical tests will be performed with a two-sided type I error rate of 5%.

### Qualitative interview analyses

All interviews with dyads allocated to the SI group will be audio-recorded, transcribed verbatim and anonymised. The interviews will be analysed by using conventional qualitative content analysis [58] and a Grounded Theory approach [59]. Qualitative analysis of the post-SI interviews will be led by the Catholic University of Applied Sciences Freiburg (CUF). Researchers at respective sites will identify initial themes in their native language. CUF will then combine the whole dataset and generate a final code list using Grounded Theory (Glaser and Strauss, 1967 [59]) methodology. This will be through an iterative process of data collection and analysis to develop initial themes, prior to analysing the entire data set and developing a model based on emerging categories. Key themes and quotations will be selected for translation into English from native languages. QDA software will be used (MaxQDA) [60] to keep transcripts and quotes in respective native languages during the whole analysis process. The participant diaries will be used by the SST to inform and shape their intervention plan. At the end of the study, the diaries will be analysed in relation to the process measures of the trial, reported in a separate article.

### Health economic analysis

A within-trial cost-effectiveness analysis will be performed. All costs consumed and quality-adjusted life-years (QALYs) gained within the 36 weeks of the trial will be calculated for both the SI group and the CAU group. Costs will be estimated on the basis of the resource use data collected in the trial and applying unit costs from country-specific reports and the published literature. The health utility scores will be multiplied by the duration of time spent on each health state to generate QALYs. The incremental cost-effectiveness ratios of the SI compared with CAU will then be calculated.

A model-based cost-effectiveness analysis will be conducted to estimate costs and effects. Parameters in the model will be specified using data collected within the trial, published literature, or expert opinion. In the analysis, the impact of parameter uncertainty will be explored in one-way sensitivity analysis on each parameter and probabilistic sensitivity analysis using a Monte Carlo simulation with 1000 iterations. A cost-effectiveness acceptability curve will be used to describe the probability that the cost per QALY gained from the analysis is cost-effective for a range of levels of willingness to pay of the decision maker (their ceiling cost-effectiveness ratio). The net benefit will be estimated at the willingness-to-pay threshold for each country, respectively.

### Study governance

#### EARB composition and role

The two main missions of the Ethical Advisory and Review Board (EARB) are to review adverse events/serious adverse events (AEs/SAEs) that may occur during the trial and to give ethical input through its independent chair. The role of the EARB is also to provide advice, through its chair, to the TSC, Trial Management Team (TMT) and any funder on the above aspects of the trial.

The members are appointed by the coordinating investigator on behalf of lead organisation of the trial (University of Manchester). Membership consists of a chair, the principal investigator (PI) of each of the five study sites, the local sponsor or representative from each site, and a representative from EUCLID.

The TSC will oversee all aspects of the design, conduct, management, reporting and dissemination of the trial. It will be composed of independent members, the chair of the EARB, site representatives, statisticians, methodologists, project coordinator and devices suppliers.

EARB and TSC will ensure the highest standards of clinical research, covering scientific quality, ethical standards and all related management issues, in compliance with GCP. The trial will adhere to GCP and standard operating procedures (SOPs) of SENSE-Cog for all trial and data management, statistical and regulatory matters. All research staff (participant facing) will undergo training in GCP (or equivalent accredited standards at their local site) with regard to the conduct of clinical trials. Trial-specific training will be delivered to all research and sensory support staff prior to the start of the study.

A TMT (chief investigator, statisticians, methodologists, project coordinator, clinical research associates, data manager and any relevant participants to discuss specific issues) will undertake the day-to-day management of the study. The EARB, TSC and TMT will regularly interact to ensure a smooth trial conduct.

### Safety

As the study is low-risk, a formal Data Monitoring Committee is not necessary. Instead, the EARB will review on a regular basis (monthly initially) AEs and SAEs and their relatedness with study intervention. Any decision to stop the trial will be made in conjunction with the TSC.

There is a small risk of falls when introducing new glasses. Therefore, the optometrist or vision specialist will introduce the glasses step-wise where necessary. Consistent and thorough checking by the SST will occur to ensure that visual devices are appropriate and, where inappropriate, will refer back to clinical services to refine the prescription. The SI up to 18 weeks may be a large commitment for some participants, so we will make clear the benefit to them and be flexible around participant availability for the SI visits.

Each local sponsor will ensure that the appropriate insurance and indemnities are adhered to in accordance with national guidelines to ensure that the highest standard of safety is maintained and that thorough safety monitoring is undertaken throughout the trial. This process will follow a trial-specific SOP for reporting AEs and SAEs. SAEs will be notified to the coordinating investigator, the local sponsor and EUCLID in accordance with a specific reporting time frame. Each local investigator and site staff will be responsible for detecting, documenting and reporting AEs or SAEs. AEs and SAEs will be reviewed on the whole during EARB meetings (initially monthly and at least every 6 months) and TSC meetings (annually). AEs and SAEs will be collected from the date the consent form is signed and up to 1 month after the planned end of participant follow-up when this could be due to the study. A phone call to both groups will assess AEs and SAEs at weeks 8 and 26. After the initial AE/SAE report, the local investigator will follow up the participant until the event has resolved or the participant is lost to follow-up. Additionally, the intensity and causality of each AE will be classified by the local site PI according to severity. The local site PI will use their clinical judgement to determine the relationship between the SI or the trial and the occurrence of each AE/SAE. In this process, the natural history of the underlying condition, concomitant treatments, other risk factors, and the temporal relationship to the AE/SAE to the study SI will be considered. The local site PI will determine whether an SAE is expected or not.

### Data management of the RCT

Different tasks of data management (from study design to database closure) and the responsibilities of each person involved in the data management process and quality control are detailed in a data management plan. Data are collected by using an eCRF (screening, baseline and follow-up data); diaries, completed by PwD and their companion (to assess whether the intervention is acceptable, tolerated, helpful and useful and to include any comments relevant to the intervention components or delivery); a logbook (SI data), completed by the SST; and qualitative interviews, audio-recorded.

Any original document or information recorded during the study is defined as a source document and the eCRF must accurately reflect the data in the source document. Source document in the framework of the study can be paper CRF, original copy of scales or medical files.

Consistency checks will be programmed by the data manager to check the consistency and the completion of data in the eCRF. The list of consistency checks will be predefined by the project team and passed on to the data manager who will write a study-specific data validation plan. Additional queries might also be raised by the clinical research assistant. Queries are sent to the clinical site via the eCRF. The data manager will complete self-evident corrections (SECs) in the database following rules defined in the SEC plan validated and signed by the sponsors and the investigators before implementation. Remote and onsite monitoring is organised throughout the trial to ensure compliance to the protocol, regulations and GCP recommendations.

### Dissemination policy

Results of the RCT will be submitted for publication in a peer-reviewed journal, and priority will be given to open-access publications, and presentations of key results will be made at local, national and international conferences in relevant fields. Feedback on study outcomes will be offered to study participants, our research user group (RUG), and the lay public by using various formats (on-line, print material, and lectures), including the SENSE-COG website (https://www.sense-cog.eu/), in all five countries involved.

#### Patient and public voice

Informed by principles of public involvement in research [61], the SI development involved a co-operative approach with “patient and public voice” (PPV) members at each stage during the field trial [27]. This was conducted with SENSE-Cog RUG in each of the study sites. Details of the PPV RUG training and contributions are outlined in a separate report [62].

### Provisions for ancillary and post-trial care

For each site, local arrangements with partner clinical services are in place to manage post-trial care; specific compensation for harm is incorporated within each local site’s sponsor agreements and liability arrangements, which differ at each site, according to the sponsoring organization.

### Local sponsors

There is no primary sponsor. Each site will have a local sponsor responsible for governance and research conduct at that site. Local sponsor details are as follows:

- Research governance sponsor representative, Manchester, UK: Lynne MacRae,
- University of Manchester, Simon Building, Oxford Road, Manchester, UK
- M13 9PL, phone: + 44(0)161275 5436, email: lynne.macrae@manchester.ac.uk;
- Local sponsor representative, Athens, Greece: Antonios Politis, 1st Department of Psychiatry, Division of Geriatric Psychiatry, Eginition Hospital National and
- Kapodistrian, University of Athens, 74 Vas. Sophias Avenue, 11,528, Athens, Greece, phone: (+ 30) 21 07 28 92 72, email: apolitis@med.uoa.gr;
- Local sponsor representative, Dublin, Ireland: Ann Dalton, St. James’s Hospital James’s Street, Dublin, Ireland, email: CEOPA@stjames.ie;
- Local sponsor representative, Nice, France: Eric Monch, University Hospital of Nice
- Cimiez Hospital, 4 avenue Reine Victoria - BP 1179, 06003 Nice Cedex 1, phone: 33 (0)4 92 03 40 11, email: monch.e@chu-nice.fr;
- Local sponsor representative, Nicosia, Cyprus: Fofi Constantinidou,
- Center for Applied Neuroscience and Department of Psychology, Kallipoleos 75, University of Cyprus, Nicosia 1678, Cyprus, phone: + 357 22 89 2078, email: fofic@ucy.ac.cy.

### Authorship eligibility guidelines and any intended use of professional writers

No professional writers are planned. Authorship will follow standard guidelines for attribution and responsibility for content and will be monitored through the TMT and then the TSC and ultimately through the full SENSE-Cog programme’s Steering Committee, which includes representation of all of the consortium partners.

### Plans for communicating important protocol modifications

In accordance with local research ethics committee/institutional review board (REC/IRB) requirements, this will be conducted under the rubric of “major” and “minor” amendments; no changes will be acted upon until the amendments have been accepted at all sites.

### Interim analyses and stopping guidelines

Since this is a very-low-risk RCT, there is no data monitoring and ethics committee (DMEC) and no interim analysis is planned for either safety or futility analysis.

### Public access to the full protocol, participant-level dataset, and statistical code

The full protocol will be communicated with primary publication of study results (and statistical code depending on the journal), and participant-level dataset (and statistical code) will be accessible through request to the EARB.

### Data transfer

The conditions for data transfer of all or part of the study database will be decided by the EARB and will be the subject of a written contract. We will deposit data on the Dementias Platform UK (DPUK) databank.

### Criteria for discontinuing or modifying allocated interventions for a given trial participant

Since this is a very-low-risk RCT, it is unlikely that the intervention will have to be discontinued, but modification of how the intervention will be delivered will be participant-specific since this is a pragmatic, tailored intervention with no specific dosing, aside from the recommended number of therapist visits. Decisions on how to modify the intervention will be taken by the therapist delivering the intervention, supervised by the senior sensory therapist in regular 1:1 supervision sessions and group oversight sessions (with all the site therapists). If any participant withdraws consent or experiences an SAE, they will be withdrawn from the study.

## Discussion

The main strength of the SENSE-Cog RCT is that it is the first trial to evaluate a complex intervention for sensory correction and support for PwD on a European scale and with a parallel process evaluation. This will enable the research team to understand results, delivery, context issues and causal mechanisms. The sample size will enable us to highlight a clinically relevant difference in DEMQOL analysis.

The main limits of the trial are the inter-country biases, which may affect the data. Furthermore, owing to the patient-reported nature of the intervention, the study is not double-blinded. We anticipate that there may be challenges to recruitment if participants do not identify themselves as having sensory impairment. There have been some time delays to setting up the project and completing the field trial [27] as a result of developing consistent processes across different European countries with respective health systems. This rigorous approach to set-up aims to set the foundation for a robust RCT.

If following trial completion the SI does demonstrate improvement in QoL, the aim is to develop a toolkit of training materials, resources and information to be available to health and social care providers to implement in routine practice. This may offer a viable therapeutic tool for sensory remediation for people living with dementia and sight or hearing loss across Europe. Finally, we aim to be able to describe the entire programme of work of the SENSE-Cog H2020 in the context of the SENSE-Cog RCT.

### Trial status

The article is based on the SENSE-Cog RCT protocol version 3.0 of 22 January 2018. The SENSE-Cog programme, of which the RCT is one work package, began on 1 January 2016. Recruitment started on 30 April 2018 in the UK, and the first participant has been recruited in the UK. The end date for the trial follow-up is planned on 31 December 2020.

## Additional file

Additional file 1: SPIRIT 2013 Checklist: Recommended items to address in a clinical trial protocol and related documents. (DOC 123 kb)

## Abbreviations

AD: Alzheimer’s disease
AE: Adverse event
BTE: Behind-the-ear
CAU: Care as usual
CRF: Case report form
CUF: Catholic University of Freiburg
DEMQOL: Dementia Quality of Life
EARB: Ethical Advisory and Review Board
eCRF: Electronic case report form
EDTRS: Early Treatment Diabetic Retinopathy Study
EUCLID: European Clinical Trials Platform & Development
GCP: Good Clinical Practice
ICD-10: 10th revision of the International Statistical Classification of Diseases and Related Health Problems
MoCA: Montreal Cognitive Assessment
PEEK: Portable Eye Examination Kit
PI: Principal investigator (at each site)
PID: Patient identifiable data
PPV: Patient and public voice
PwD: Person/people with dementia
QALY: Quality-adjusted life-year
QoL: Quality of life
RCT: Randomised controlled trial
RUD-Lite: Resource Utilization in Dementia-Lite
RUG: Research user group
SAE: Serious adverse event
SAP: Statistical analysis plan
SEC: Self-evident correction
SI: Sensory intervention
SOP: Standard operating procedure
SST: Sensory support therapist
TMT: Trial management team
TSC: Trial steering committee
VAD: Vascular dementia
W: Week/s
